# A scoping review, novel taxonomy and catalogue of implementation frameworks for clinical decision support systems

**DOI:** 10.1186/s12911-024-02739-1

**Published:** 2024-11-01

**Authors:** Jared M. Wohlgemut, Erhan Pisirir, Rebecca S. Stoner, Zane B. Perkins, William Marsh, Nigel R.M. Tai, Evangelia Kyrimi

**Affiliations:** 1 Centre for Trauma Sciences, Blizard Institute, Queen Mary University of London, London, UK; 2 Royal London Hospital, Barts Health NHS Trust, London, UK; 3 School of Electronic Engineering and Computer Science, Queen Mary University of London, Mile End Road, London, E1 4NS UK; 4 Royal Centre for Defence Medicine, Birmingham, UK

**Keywords:** Implementation, Clinical decision support system, Frameworks, Design, Development, Evaluation, Implementation, Adoption, Scoping review

## Abstract

**Background:**

The primary aim of this scoping review was to synthesise key domains and sub-domains described in existing clinical decision support systems (CDSS) implementation frameworks into a novel taxonomy and demonstrate most-studied and least-studied areas. Secondary objectives were to evaluate the frequency and manner of use of each framework, and catalogue frameworks by implementation stage.

**Methods:**

A scoping review of Pubmed, Scopus, Web of Science, PsychInfo and Embase was conducted on 12/01/2022, limited to English language, including 2000–2021. Each framework was categorised as addressing one or multiple stages of implementation: design and development, evaluation, acceptance and integration, and adoption and maintenance. Key parts of each framework were grouped into domains and sub-domains.

**Results:**

Of 3550 titles identified, 58 papers were included. The most-studied implementation stage was acceptance and integration, while the least-studied was design and development. The three main framework uses were: for evaluating adoption, for understanding attitudes toward implementation, and for framework validation. The most frequently used framework was the Consolidated Framework for Implementation Research.

**Conclusions:**

Many frameworks have been published to overcome barriers to CDSS implementation and offer guidance towards successful adoption. However, for co-developers, choosing relevant frameworks may be a challenge. A taxonomy of domains addressed by CDSS implementation frameworks is provided, as well as a description of their use, and a catalogue of frameworks listed by the implementation stages they address. Future work should ensure best practices for CDSS design are adequately described, and existing frameworks are well-validated. An emphasis on collaboration between clinician and non-clinician affected parties may help advance the field.

## Background

The amount of knowledge available to clinicians to make evidence-based decisions is growing rapidly [1]. Clinicians make myriads of decisions daily. Methods of offloading analytical processes could reduce cognitive load while improving clinician situational awareness. Many clinical decision support systems (CDSS) have been developed to help clinicians in their decision-making [2]. According to Shortliffe and Cimino, CDSS provide patient-specific recommendations based on clinical scenarios, which usually follow clinical rules and algorithms, a cost–benefit analysis, or clinical pathways [3]. The National Academy of Medicine states that CDSSs form a key component of a learning health system, which can assist the implementation and adoption of new knowledge into practice [4]. Further, CDSSs are deemed a “practical necessity for every clinician in our rapidly evolving health and healthcare landscape”, as they can “ameliorate the burden that exponentially expanding clinical knowledge as well as care and choice complexity place on the finite time and attention of clinicians, patients, and every other member of the care team” [5].

Despite an increased research effort to develop complex and accurate CDSS and to understand potential clinical benefit, CDSS are still not widely implemented into clinical practice [6, 7]. Recent studies have focused on investigating the barriers and facilitators for adopting CDSS [8–10]. From an organisational standpoint, barriers to adoption include human–computer interaction issues [11–15], timing of use [11, 16–18], lack of training [11, 16], lack of integration into the clinical workflow [13], CDSS inefficiencies [6, 12, 16], and concerns regarding clinicians’ autonomy [12–14]. Adoption is also adversely affected by professional resistance to change [12, 19], and lack of end-user involvement in CDSS development and evaluation [13, 20].

Many implementation frameworks have been published to overcome the identified barriers to adoption and offer guidance to enable the effective implementation of CDSS in clinical practice [21]. Notably, implementation frameworks differ in complexity. For instance, some of the published frameworks are ‘whole system’ [22], while some focus on only one or more aspects of the adoption process, such as design [23] or acceptance [24]. There is a significant overlap among the published frameworks, yet each miss key domains included in others [25]. Some studies have reviewed a limited number of key frameworks with the intent to unify these into an overarching whole [26]. Other studies have reviewed existing frameworks and explored barriers and facilitators to identifying and selecting the appropriate implementation frameworks [21]. Sifting through and making sense of such a large pool of implementation frameworks is challenging. To our knowledge, no previous study has aimed to providing a taxonomy of these frameworks. Our scoping review aims to help researchers better navigate the existing literature on frameworks for guiding the process from concept to adoption of CDSS. We believe this is of benefit to co-developers (researchers in both computer science and medicine) who are interested in implementing CDSS in healthcare.

The primary objective of this scoping review is to synthesise key domains and sub-domains described in existing CDSS implementation frameworks from concept to adoption into a novel taxonomy and demonstrate most-studied and least-studied domains. The studied CDSS implementation frameworks have either specifically designed or applied to CDSSS. A secondary objective is to describe the frequency and manner of use of included frameworks, as well as academic impact. A tertiary aim is to catalogue existing CDSS implementation frameworks by implementation stage.

## Methods

### Literature search

A search of major health and health informatics literature databases including Pubmed, Scopus, Web of Science, PsycInfo and EmBase was conducted on 12/01/2022, limited to English language, between 2000–2021, and selecting only papers where the described keywords were present in their title or abstract. Our keywords contained three main terms. The first term was *clinical*, which indicated the discipline that we were interested in. The second term was *decision support system*, which is the class of system that we targeted for our review. The third term was *adoption framework*, which was the focus of our review. A set of synonyms often found in relevant literature were also searched. The synonyms were based on an initial pilot study, during which a small number of key papers on CDSS implementation stages were reviewed and the main terms used were identified. The full list of terms and synonym can be found in Table 1.

**Table 1:** Search terms and synonyms

Terms	Synonyms
Clinical	clinician, physician, healthcare
Decision support	decision aid, prediction rule
System	tool, technology
Adoption	adopt, implementation, implement
Framework	guideline, theory

The searches were restricted to the English language, including the years 2000-2021, and keywords within the Title or Abstract. The search string was ((clinical OR clinician OR physician OR healthcare) AND ("decision support" OR "decision aid" OR “prediction rule”) AND (system OR tool OR technology) AND (adoption OR implementation OR adopt OR implement) AND (framework OR guideline OR theory))

Additional screening was conducted to exclude papers that did not permit access to the full paper and were not journal or conference peer-reviewed articles. The reason for excluding non-peer-reviewed work is that we wanted to ensure that included frameworks have been properly scrutinised. The reason for excluding papers published before 2000 was that we wanted to ensure the CDSS frameworks would be relevant for computer-based modern and future healthcare systems. The authors’ area of academic interest is artificial-intelligence models, which have largely failed to be adopted into clinical practice [6, 7]. Therefore, we wanted to exclude frameworks published prior to widespread existence of computerised health systems, which may not be relevant. The remaining papers were those adhering to the inclusion (IC) and exclusion criteria (EC) presented in Table 2. Papers were included if they introduced a new framework, or either extended, integrated or validated an existing framework. We considered an existing framework ‘validated’ if it was used and objective results were presented to demonstrate how it was used. We considered the ‘evaluation’ of a framework to refer to whether the CDSS had an effect on behaviour change and process metrics of uptake, including reach, adoption and maintenance, rather than in a computational sense of AI models referring to its predictive performance.

**Table 2:** Inclusion and exclusion criteria

Inclusion criteria (IC)	
IC-1:	Propose a new framework that describes at least one aspect of the process from concept to adoption of CDSS in practice
IC-2:	Propose an extension of an existing framework that describes at least one aspect of the process from concept to adoption of CDSS in practice
IC-3:	Propose a framework with the intend to assist implementation of healthcare technologies, where CDSS has been mentioned as a part of it
IC-4:	Implement an existing framework in healthcare settings
Exclusion Criteria (EC)	
EC-1:	Framework not developed or implemented in healthcare
EC-2:	Healthcare related but not specific to CDSS or technology (e.g. surgical interventions, drugs etc.)
EC-3:	Framework proposed but not related to a specific stage from concept to adoption of CDSS in practice
EC-4:	There is a statement in the title/abstract that a framework is provided, but none is detailed
EC-5:	Publication limited to adoption challenges, suggestions, recommendations, or facilitators without providing a framework
EC-6:	Review of existing frameworks without proposition of novel frameworks or study of implementation into healthcare settings

Two additional search approaches were used; (1) pursuing references of references (“snowballing”), and (2) expert inquiry to identify relevant papers. This was done to include relevant papers that might not have been captured due to missing keywords in the title and abstract. Papers identified were subject to IC/EC described in Table 2.

To ensure a consistent review process, a training session was conducted during which three papers were reviewed by the four reviewers (JW, RS, EP and EK) involved in this study, followed by a thorough discussion on the given answers and how they were derived. A dual independent assessment of each paper was conducted in two successive review rounds. Each paper was reviewed by one clinician and one non-medical researcher / computer scientist. In cases where responses differed, the four reviewers worked collaboratively and resolved the conflict by consensus.

### Study aims

This study has three main aims, and for each aim, a set of research questions (RQ) were addressed. The aims, rationale, and related RQs are presented in Table 3.

**Table 3:** Study aims and research questions

Aim	Rationale	Research question (RQ)
Frameworks descriptive taxonomy	Synthesise the domains and sub-domains of published CDSS implementation frameworks into a taxonomy, and describe the most studied and least-studied domains	RQ1: Which are the most discussed domains in the published frameworks? RQ2: Which domains have been least-discussed in the published frameworks?
Frameworks academic application	Describe how the published frameworks have been used in academic practice	RQ3: What is the most popular published framework and how has it been used in healthcare settings? RQ4: What is the academic impact of these frameworks?
Frameworks co-developers catalogue	Describe how the published frameworks fit within each stage from design to adoption	RQ5: How do the published frameworks map within the overarching implementation process from design to adoption?

### Implementation stages

During an initial pilot study, a small number of papers on CDSS implementation stages were reviewed. Each reviewer independently proposed a categorization of the implementation stages from design to adoption of CDSS. The proposed stages were combined, and disagreements were resolved by discussion. Upon consensus, four main implementation stages were identified as described in Table 4.

**Table 4:** Stage of implementation

Stage	Description
Design & Development	Clarification of who, why, how the CDSS is intended to be used; the development process
Evaluation	Predictive performance of CDSS; initial impact study; feasibility study
Acceptance & Integration	User acceptance; usability; integration/ implementation into existing workflow
Adoption & Maintenance	Dissemination, surveillance, and monitoring; revisions; organisation reliance to adaptations; follow-up study

### Data extraction

A data extraction sheet was created and piloted by all reviewers on Microsoft Excel (Microsoft Corp, Redmond, WA, USA), sharing via Microsoft Teams. Data extraction was divided equally between four reviewers, and each paper double-checked. Attributes extracted for each included paper included: its definition for CDSS (if provided); which stage(s) of implementation the framework addressed (design and development; evaluation; implementation/integration; adoption/maintenance); and for each stage(s) of implementation the framework addressed, we collected domain names and definitions (main parts of each framework), and sub-domain names and definitions (sub-part of each framework). This scoping review was reported using the preferred reporting items for systematic reviews and meta-analyses extension for scoping reviews (PRISMA-ScR) [27].

### Descriptive taxonomy

A descriptive taxonomy (Aim 1) of domains and sub-domains described by published CDSS implementation frameworks was created. Domains and sub-domains from frameworks which were novel, extensions of previous CDSS frameworks, or integrated into previous CDSS frameworks, were included. Two authors (one clinician and one non-medical researcher / computer scientist) independently identified which implementation stage(s) each framework addressed (Table 4) and extracted all domains and related sub-domains. Each domain and sub-domain within each implementation stage was then examined by two authors independently (JW and EK), and similar categories were merged/combined, to form more a truncated but inclusive list. Least-discussed and most-discussed domains were then quantified and graphically displayed.

### Frameworks academic application

The frequency of frameworks described in included studies (Aim 2) was quantified by adding the number of papers which reported validation of a published framework and was graphically displayed. As a sensitivity analysis of the academic impact of each framework, a bibliometric search of citations of the original publication introducing each framework was conducted. Search engines explored included Web of Science and Google Scholar (searched 7th September 2022).

### Frameworks co-developers catalogue

The final aim of the study (Aim 3) was addressed by mapping studies which propose a new framework, or extend or integrate an existing framework, within implementation stages. These frameworks are categorised based on the authors’ discipline and provide a catalogue of published CDSS implementation frameworks by stage of implementation.

## Results

### Literature search results

**Fig. 1:**
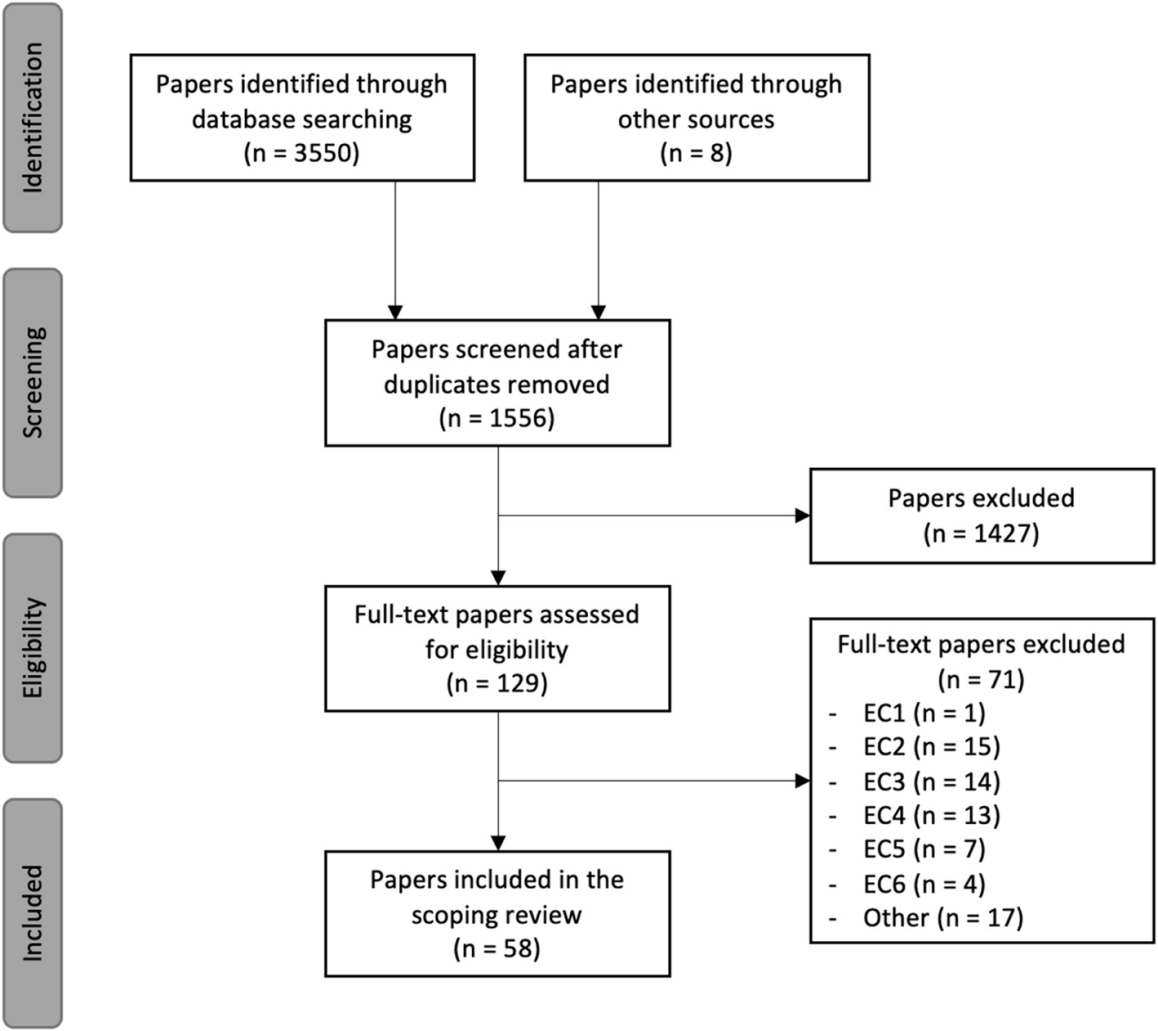
PRISMA diagram for literature selection

The number of papers identified in each database are presented in Table 5. The results of the literature selection process are presented in Fig. 1. Initially, 3558 papers were identified for screening. After removing duplicates, the abstracts were read by two reviewers and we excluded those that did not follow the IC mentioned in Table 1, resulting in 129 papers. From these, 58 papers remained for inclusion in this scoping review (Table 6). Of these 58 included papers, 21 proposed new CDSS implementation frameworks, 5 extended previous frameworks, 2 integrated into an existing framework, and 39 validated a previously developed CDSS implementation framework.

**Table 5:** Search results by database source

Database	Identified papers	Fields	Filters
Pubmed	710	Title/ Abstract	English Language, Period 2000–2021
Scopus	1240	Title/ Abstract	English Language, Period 2000–2021, Journal Type
Web of Science	1086	Title/ Abstract	English Language, Period 2000–2021, Journal Type
PsycInfo	100	Title/ Abstract	English Language, Period 2000–2021, Journal Type
Embase	414	Title/Abstract	English Language, Period 2000–2021, Journal Type

**Table 6:** Characteristics of included studies (*n* = 58)

Ref	First Author, Year	Country^a^	Authors discipline	Framework name	Study Type	Description of framework development method	Framework users
[26]	Camacho, 2020	USA	Β	BEAR	N; V	Mapped constructs from TDF and UTAUT	Researchers
[28]	Khong, 2015	Singapore	M	N/A	N	Literature review of existing frameworks	N/A
[29]	Kannry, 2015	USA	M	iCPR^c^	N	Usability testing during randomized controlled trial. Used “lessons learnt” to form a framework	N/A
[30]	Tsopra, 2021	France	Β	Ν/Α	N	No clarification beyond “The ITFoC consortium has designed a framework”	N/A
[31]	Marcial, 2019	USA	^b^	CFIR	V	No development	N/A
[32]	Liu, 2021	USA	NM	UTAUT	V	No development	N/A
[33]	Aljarboa, 2019	UK	NM	UTAUT & TTF	N, I	Used UTAUT and TTF models to create conceptual framework of factors influencing CDSS acceptance. Tested this in semi-structured interviews	General practitioners
[34]	Yusof, 2008	Malaysia	NM	HOT-fit	N; V	Literature review on HIS and IS evaluation studies and pilot testing of developed framework	Researchers & practitioners
[35]	Masterson, 2018	USA	B	RE-AIM	V	No development	N/A
[36]	Elwyn, 2008	UK	M	NPM	V	No development	N/A
[9]	Klarenbeek, 2020	The Netherlands	M	CFIR	V	No development	N/A
[37]	Paulsen, 2019	Norway	M	CFIR	V	No development	N/A
[38]	Harry, 2019	USA	M	CFIR	V	No development	N/A
[39]	Wright, 2010	USA	M	N/A	N	Discussion with a panel of CDSS experts. Results were analysed using a grounded theory method to elicit themes and best practices	Developers, implementers, and users
[40]	Greenhalgh, 2017	UK	B	NASSS	N	Literature review. Qualitative interviews, analysis of documents, ethnography, and video recording of both ends of remote consultations	N/A
[41]	Wannheden, 2017	Sweden	B	UTAUT	V	No development	N/A
[42]	Saleem, 2009	USA	B	N/A	N	Literature review	Researchers, developers, implementers, and evaluators
[43]	Lehmann, 2018	USA	B	N/A	N	Literature review	Developers
[44]	Khalifa, 2019	Australia	M	GRASP	N; V	Literature review. GRASP builds on widely accepted concepts, such as Friedman and Wyatt’s evaluation approach and the GRADE system	Expert users
[45]	Cresswell, 2020	UK	NM	TPOM	N	Literature review and qualitative data from formative evaluations of interventions	Implementers and evaluators
[46]	Craig, 2008	UK	M	MRC Framework on Evaluating Complex Interventions	E	Workshop to consider how to update framework	Health service
[47]	Li, 2020	USA	M	CFIR & RE-AIM	V	No development	N/A
[48]	Simione, 2020	USA	M	CFIR	V	No development	N/A
[49]	Singer, 2021	USA	B	N/A	N	Qualitative interviews with CDSS developers and users	Practitioners
[50]	Shapiro, 2005	USA	M	N/A	N; V	Based on literature review	Nurses
[51]	Khalifa, 2020	Australia	NM	GRASP	V	No development	N/A
[52]	Shibl, 2013	Australia	B	Extend UTAUT for GPs	E; V	Qualitative interviews with CDSS users	General Practitioners
[25]	Damschroder, 2009	USA	M	CFIR	N	Literature review. Combined constructs across published theories	Implementation researchers
[53]	Campbell, 2000	Canada	M	HITET	N	Workshop of experts to design framework	N/A
[22]	Wallace, 2011	Ireland	M	N/A	N	Literature review and collective experience of international working group	Academic developers
[54]	Sockolow, 2015	USA	M	HITREF	N; V	Literature review	Clinicians
[55]	Peleg, 2018	Israel	B	IDEAS	E	Extended the Ideation phase of IDEAS framework	N/A
[56]	Minian, 2020	Canada	M	RE-AIM	V	No development	N/A
[57]	Shah, 2021	USA	B	RE-AIM	V	No development	N/A
[58]	Short, 2021	USA	M	RE-AIM	V	No development	N/A
[59]	Fortney, 2012	USA	M	RE-AIM	V	No development	N/A
[60]	Wu, 2019	USA	M	RE-AIM	V	No development	N/A
[61]	Boateng, 2021	USA		CFIR	V	No development	N/A
[62]	Trinkley, 2020	USA	B	PRISM	V	No development	N/A
[63]	Prakash, 2021	India	O	UTAUT & Status Quo Bias & Technology Trust	I; V	Mixed-methods approach	N/A
[64]	Harry, 2020	USA	M	CFIR	V	No development	N/A
[65]	Haun, 2021	USA	M	CFIR	V	No development	N/A
[66]	Yu, 2019	Canada	M	RE-AIM	V	No development	N/A
[67]	Paulsen, 2021	Norway	B	RE-AIM	V	No development	N/A
[68]	Westafer, 2020	USA	M	Used CFIR and TDF	V	A qualitative study using CFIR and TDF for barriers and facilitators of a tool	N/A
[69]	Roebroek, 2020	Netherlands	M	UTAUT	V	No development	N/A
[70]	Sward, 2008	USA	B	N/A	E; V	Modified IT implementation framework by adding detailed constructs applicable to our analysis	N/A
[24]	Khairat, 2018	USA	M	UASAD & IPOE	N	Literature review of 14 CDSS use adoption papers, and task analysis	N/A
[71]	Ash, 2012	USA	M	Multiple Perspectives	E	No development	N/A
[72]	Van de Velde, 2018	Norway	O—Public health	GUIDES	N	Systematic literature review, a synthesis of the factors, and pilot testing of the checklist (on systematic review of CDSS trials, and focus groups)	N/A
[73]	Patel, 2020	USA	M	RE-AIM	V	No development	N/A
[74]	Abimbola, 2019	Australia	M	NASSS	V	No development	N/A
[75]	Russell, 2015	USA	M	CFIR	V	No development	N/A
[76]	Bakken, 2009	USA	M	RE-AIM	V	No development	N/A
[77]	Pannebakker, 2019	UK	M	CFIR	V	No development	N/A
[78]	Vasudevan, 2020	USA	M	CFIR	V	No development	N/A
[79]	Bean, 2021	USA	M	CFIR	V	No development	N/A
[80]	Liberati, 2017	UK	M	N/A	N	Qualitative study conducted as part of a series of randomized controlled trials of CDSSs. Used a constant comparative approach to develop a framework for guiding implementation	Doctors and nurses

*BEAR *Behaviour and Acceptance Framework, *iCPR *integrated clinical prediction rule, *CFIR *Consolidated Framework for Implementation Research, *HITET *Health Information Technology Evaluation Toolkit, *UTAUT *Unified Theory of Acceptance and Use of Technology, *TTF *task-technology fit model, *HOT-Fit *Human, Organisation, and Technology-fit Framework, *RE-AIM *Reach, Efficacy, Adoption, Implementation, and Maintenance framework, *NPM *normalization process model, *NASSS *nonadoption, abandonment, scale-up, spread, and sustainability, *GRASP *Grade and Assess Predictive tools, *TPOM *Technology, People, Organizations, and Macroenvironmental factors, *MRC *Medical Research Council, *HITREF *Health Information Technology (HIT) Reference-based Evaluation Framework, *IDEAS *Integrate, Design, Assess, and Share, *PRISM *Practical Robust Implementation and Sustainability Model, *TDF *Theoretical Domains Framework, *UASAD *the user acceptance and system adaptation design model, *IPOE *the input-process-output-engage model, *GUIDES *Guideline Implementation with Decision Support, *M *medical clinicians, *NM *non-medical researchers / computer scientists, *B *both medical clinicians and non-medical researchers / computer scientists, *O *other, *N *new framework, *E *extension to an existing framework, *I *integration of an existing framework, *V *validation of an existing framework, *N/A *not applicable

^a^Country of first author

^b^“RTI International” is a research institute

^c^iCPR is the name of their own CDS tool, integrated clinical prediction rule, not exactly a usability framework name

### Study results

This section presents results from the analysis of the included literature with respect to the objective and RQs presented in Table 3.

#### Frameworks descriptive taxonomy

**Fig. 2:**
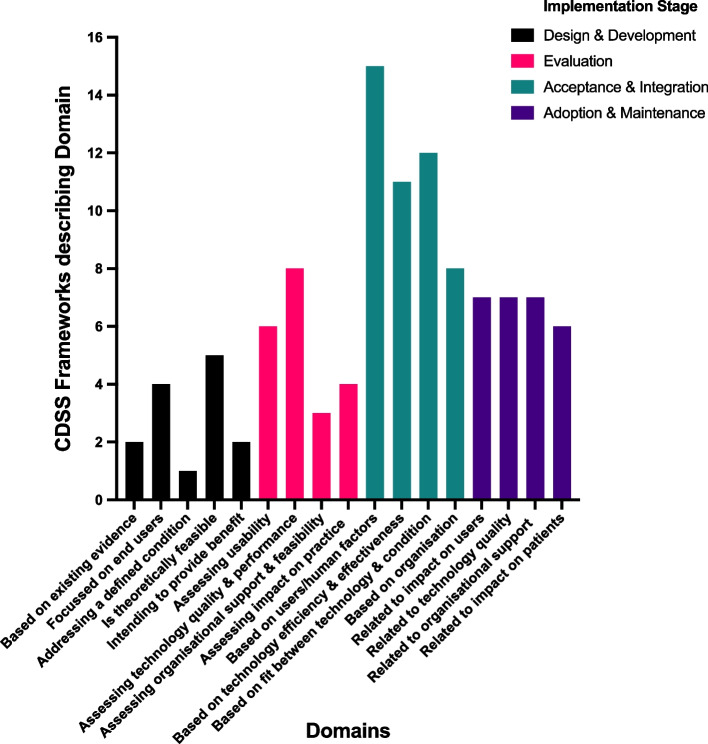
Frequency of domains by implementation stage

To address the first study objective (Table 3; RQ1 and RQ2), we created a taxonomy of the main domains and sub-domains of each new, extended or integrated CDSS implementation framework, as well as how they fit within each implementation stage (Table 7). Papers included in this scoping review that simply validated an already published CDSS implementation framework were not included in Table 7. This addresses RQ1 and RQ2, by demonstrating the most- and least-discussed domains in published frameworks. A histogram of the frequency of each domain as mentioned in published frameworks is also shown in Fig. 2. The implementation stage observed with the greatest frequency was ‘Acceptance & Integration’; within this phase, the ‘Based on user/human factors’ domain was the most popular area for frameworks to focus upon. The least frequently described implementation stage was ‘Design & Development’; and the least discussed domain was ‘Addressing a defined condition’ (Fig. 2).

**Table 7:** A descriptive taxonomy of domains and sub-domains identified in CDSS implementation frameworks which were new, an extension or integration

Implementation Stage	Domains	Sub-Domains
Design & Development	1.Based on existing evidence [46, 55] 2.Focused on end-users [40, 42, 49, 71] 3.Addressing a defined condition [40] 4.Is theoretically feasible [40, 42, 53, 71] 5.Intending to provide benefit [40, 46]	1a. Defined evidence base [46] 1b. Fits to existing workflow [55] 2a. Addresses clinical User needs [40, 42, 49, 71] 2b. Clinically appropriate input & output [40, 49, 71] 3a. Defined nature of condition or illness [40] 3b. Influence of comorbidities, socio-cultural influences [40] 4a. Data is available [40, 49, 71] 4b. Knowledge from CDSS is needed [40] 4c. Model development [40, 42, 53] 5a. Intended benefit to healthcare providers, patients, and/or affected parties [40, 46]
Evaluation	1.Assessing usability [30, 34, 43–46] 2.Assessing technology quality and performance [22, 30, 34, 43–45, 50, 54] 3.Assessing organisational support and feasibility [22, 45, 53] 4.Assessing impact on practice [22, 46, 50, 54]	1a. Actual versus intended use in target population [30, 34] 1b. System usability/user satisfaction [34, 44, 45] 1c. System explainability (explanation, debiasing, addressing uncertainty, sensibility, comprehensibility) [30, 43] 1d. Understanding the change process [46] 2a. System quality and functionality (hardware & software adaptability, flexibility, dependability) [34, 45, 54] 2b. Data availability, integrity and safety (confidentiality, quality, privacy and security) [30, 34, 45, 54] 2c. Predictive performance (discrimination, calibration, thresholds) [22, 30, 43] 2d. Validation (internal, temporal, external) [44, 50] 3a. Organisational support, structure, security and environment [45] 3b. Feasibility of impact study [22, 53] 4a. Impact on practice (effectiveness on clinically relevant outcomes) [22, 46, 50, 54]
Acceptance & Integration	1.Based on users/human factors (inner setting) [8, 24–26, 28, 29, 33, 40, 42, 44, 45, 52, 54, 70, 72, 80] 2.Based on technology efficiency and effectiveness [24–26, 28, 33, 42, 45, 54, 55, 70, 72] 3.Based on fit between technology and condition [25, 26, 28, 33, 42, 44, 45, 52, 55, 70, 72, 80] 4.Based on organisation and wider system (outer setting) [24–26, 33, 45, 54, 70, 80]	1a. Usability, usefulness, trustworthiness, performance expectancy, user expectations and needs [24–26, 28, 33, 44, 52, 54, 72, 80] 1b. User mental effort, situational awareness, memory, attention, and decision-making [26, 29, 42] 1c. User professionalism (knowledge, skills/abilities, role/identity, intentions/goals) [26, 70] 1d. User personality (beliefs, attitudes, emotions, behavioral regulation) [26, 70] 2a. System efficiency, accessibility, adaptability [25, 26, 33, 45, 54] 2b. System communicability (interface design, informativeness) [25, 33, 40, 42, 70, 72] 2c. Informatics and patient safety (privacy, security, risk) [24, 26, 42, 45] 3a. Workflow integration, clinicians familiarised with technology, relevance, training required [26, 28, 42, 45, 52, 55, 72, 80] 3b. Perceived benefits/value and consequences (relative to standard care) [25, 26, 28, 33, 70] 3c. Effects on quality, effectiveness of care, safety, clinician autonomy, healthcare efficiency [26, 44] 4a. Professional and social influences and structures (culture) [25, 26, 44, 45, 70] 4b. Organisational support (IT systems, environmental context and resources) [25, 26, 33, 54] 4c. Capacity to innovate; readiness for change; organisational resilience [25] 4d. Political, policy, regulatory, legal landscape [24, 26, 45, 80]
Adoption & Maintenance	1.Related to impact on users [25, 28, 39, 49, 54, 63, 71] 2.Related to technology quality [22, 25, 28, 39, 49, 54, 71] 3.Related to organisational support [25, 39, 40, 44, 53, 54, 71] 4.Related to impact on patients [22, 28, 39, 44, 46, 54]	1a. User familiarity, motivation, intention [28] 1b. Impact on user decisions, workflow, needs, role, profession, identity [28, 39, 49, 71] 1c. Social/cultural influence [25, 28, 63] 2a. Barriers/facilitators of technology (usability, usefulness, relevance, trust, complexity, hardware/software) [22, 25, 28, 54] 2b. Maintain/monitor use, quality, data completeness [39, 49, 54, 71] 2c.Verify predictive performance, acceptability [22] 2d. Update/revise model [39, 49] 2e.Relative benefits (efficiency, effectiveness) [25] 3a. Organisational governance, policies, support, motivation and incentives [25, 39, 40, 53, 71] 3b. Cost effectiveness [25, 44, 54] 4a. Monitor/surveillance of clinical impact (effectiveness, long-term follow-up) [39, 46] 4b. Patient satisfaction, privacy, safety, preferences [28, 44, 54]

#### Frameworks academic application

The second study objective (Table 3; RQ3 and RQ4) was addressed by studying how many included studies reported implementation of a published framework. Use of a published framework was described in 39 out of the 58 papers included in the scoping review. Three main purposes of framework use were identified:

1. To evaluate adoption: using a CDSS framework to evaluate the adoption and acceptance of a specific CDSS.
2. To understand attitudes toward implementation: creation of an interview protocol and/or analysis results to better understand the barriers and facilitators of CDSS adoption.
3. To validate a framework: assess a framework’s validity through case studies and/or interviews.

**Fig. 3:**
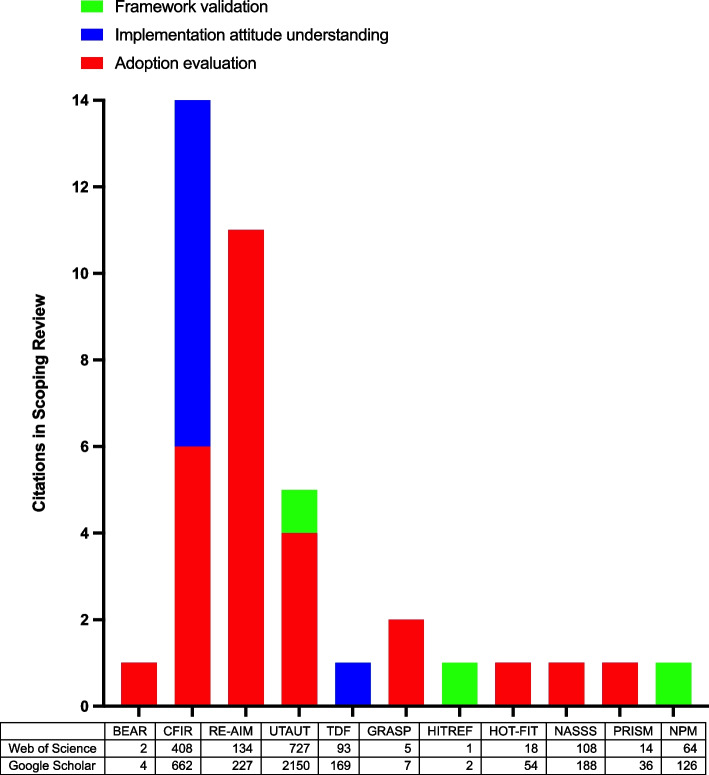
Frequency and academic use of published adoption frameworks in scoping review. Legend: The graph represents frequency of use and type of academic use within included studies in this review. The table represents the average number of annual citations since publication of each framework to 2022, in each key search engine (Web of Science and Google Scholar). Frameworks included in our reference list were BEAR (Behaviour and Acceptance Framework; Camacho 2020) [26], CFIR (Consolidated Framework for Implementation Research; Damschroder 2009) [25], GRASP (Grade and Assess Predictive tools; Khalifa 2019) [44], HITREF (Health Information Technology (HIT) Reference-based Evaluation Framework; Sockolow 2015) [54], HOT-FIT (Human, Organisation, and Technology-fit Framework; Yusof 2008) [34], and NASSS (Nonadoption, Abandonment, Scale-up, Spread, and Sustainability; Greenhalgh 2017) [40]. Original frameworks not included in the scoping review included those for RE-AIM (Reach, Efficacy, Adoption, Implementation, and Maintenance framework; Glasgow 1999) [81], UTAUT (Unified Theory of Acceptance and Use of Technology; Venkatesh 2003) [82], TDF (Theoretical Domains Framework; Michie 2005) [83], PRISM (Practical Robust Implementation and Sustainability Model; Aqil 2009) [85], and NPM (Normalization Process Model; May 2009) [84]

As shown in Fig. 3, the most frequently used or reported frameworks in the studied literature were the Consolidated Framework for Implementation Research (CFIR) [25], and the Reach, Efficacy, Adoption, Implementation, and Maintenance framework (RE-AIM) [81]. The most frequent use of the framework was to evaluate adoption. Regarding the two most popular adoption frameworks, CFIR was used mostly for understanding attitudes towards implementation [9, 37, 38], while RE-AIM was used solely to evaluate adoption [58, 60, 73]. The sub-table in Fig. 3 shows the average annual citations since publication of each framework in key search engines (Web of Science and Google Scholar). Using this metric, the frameworks which have had the most academic impact were UTAUT (Unified Theory of Acceptance and Use of Technology) [82], followed by CFIR [25], RE-AIM [81], NASSS (Nonadoption, Abandonment, Scale-up, Spread, and Sustainability) [40], TDF (Theoretical Domains Framework) [83], NPM (Normalization Process Model) [84], HOT-FIT (Human, Organisation, and Technology-fit Framework) [34], PRISM (Practical Robust Implementation and Sustainability Model) [85], GRASP (Grade and Assess Predictive tools; Khalifa 2019) [44], BEAR (Behaviour and Acceptance Framework) [26], and HITREF Health Information Technology (HIT) Reference-based Evaluation Framework; Sockolow 2015) [54] (Fig. 3).

#### Frameworks co-developers’ catalogue

**Fig. 4:**
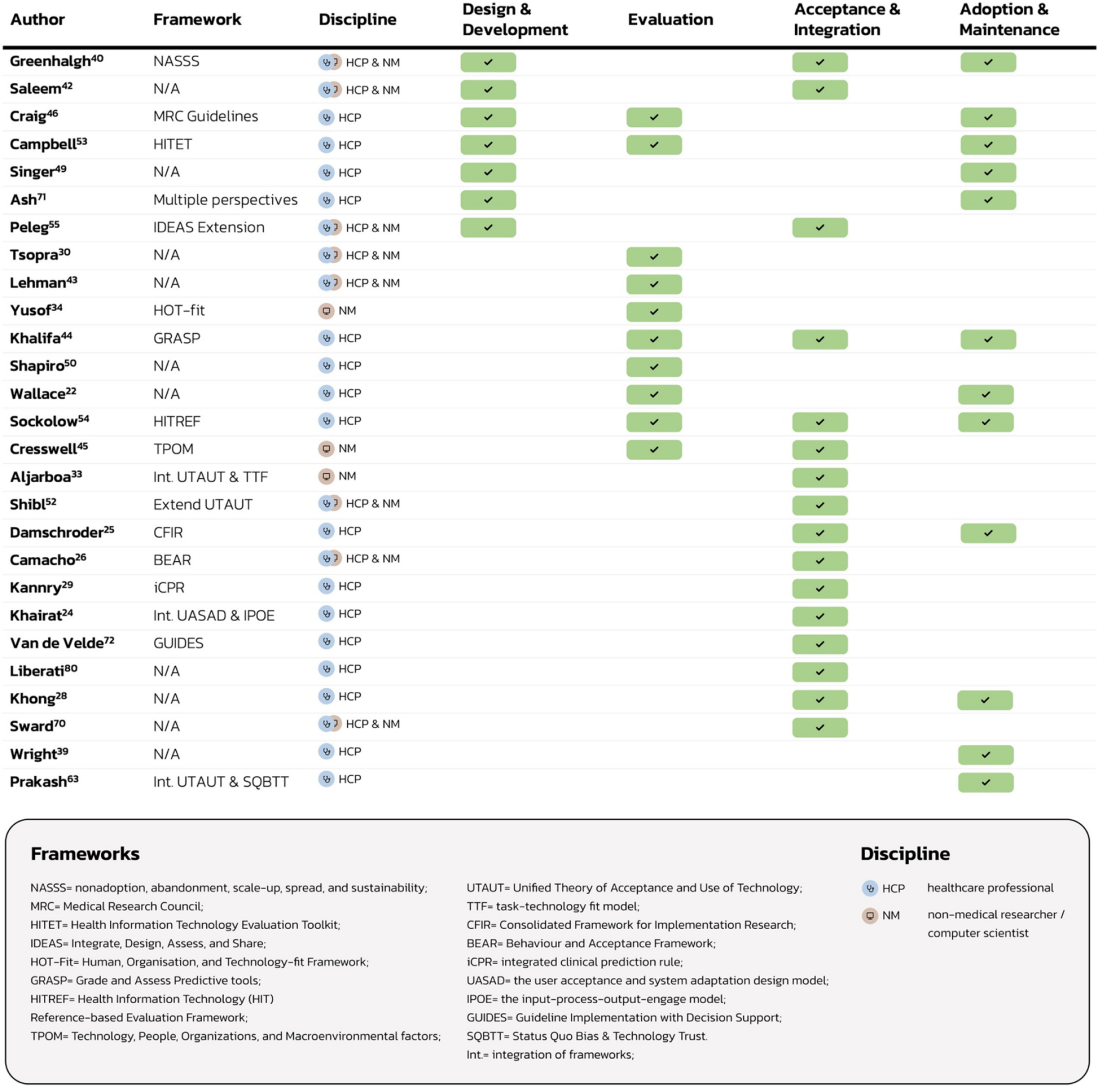
Co-developers catalogue of published frameworks by related implementation stage

The third study objective (Table 3; RQ5) was addressed by studying how published frameworks mapped within the overarching implementation process from design to adoption. The implementation stage that has gained less research attention is the initial ‘Design and Development’ phase (Fig. 4). Many frameworks address multiple implementation stages, including five frameworks which are address three implementation stages, though no framework addressed all four implementation stages. Most of the published frameworks have been created by healthcare professionals without any collaboration with non-medical researchers / computer scientists.

## Discussion

### Findings and strengths

This scoping review has confirmed a lack of standardisation of frameworks which have been developed to aid implementation of clinical decision support systems. Multiple frameworks have been developed, each focussing on one or many stages of implementation, but few in a holistic manner which could guide co-developers through their project from design to adoption. Most frameworks have been created without any subsequent attempt at validation in academic or clinical practice. In this context, instead of creating a new framework to add to a crowded market, we have instead attempted to make sense of the existing literature, in order to best serve co-developers by helping them decide which framework(s) best suits their needs. We have therefore provided a novel taxonomy of domains included in existing CDSS implementation frameworks, in order to identify domains which have been most- and least-addressed; quantified the frequency of use of these frameworks within included studies, along with a bibliometric sensitivity analysis, to identify frameworks which have gained the most popularity among researchers; and provided a practical catalogue from which co-developers can select existing frameworks which best suit their project. To our knowledge, this is the first study to provide a taxonomy of domains of CDSS implementation frameworks, as well as a catalogue of these frameworks. We utilised a well-structured review process, including a clear objective and related research questions, training of all study reviewers prior to conducting the review, and two rounds of review, ensuring that both a non-medical researcher / computer scientist and a clinician reviewed each paper eligible for full-text review.

In the ‘Acceptance and Integration’ stage of implementation, the most frequent domain was “Based on user/human factors”, including a sub-domain related to usability/usefulness (*n* = 10). Usability is known to be one of the main barriers to implementation and adoption of CDSS, so it is aptly reported with greater frequency in published frameworks. Usability has been defined by the International Organisation for Standardisation (ISO 9241–11:2018), as the “extent to which a system, product or service can be used by specified users to achieve specified goals with effectiveness, efficiency and satisfaction in a specified context of use” [86]. Usability can be evaluated in multiple ways, including both quantitative and qualitative methods [87]. The ‘Design and Development’ stage was less frequently studied. Only one framework focused on the condition and comorbidities which the CDSS is meant to address [40]. In addition, only one framework each focuses on the existing evidence base [46], and current workflow [55] during the design and development stage. These aspects may need more emphasis in CDSS frameworks, especially because much effort is placed on matching the technology with clinical need in later implementation stages. Of the known adoption barriers mentioned in the introduction, all were addressed within the frameworks at one of the implementation stages, however involvement of the user in the design and development stage was lacking.

This study has also highlighted how published CDSS frameworks were used in included studies (Fig. 3). The most used frameworks were found to be CFIR and RE-AIM. CFIR was used more broadly, for both evaluation of adoption and to understand attitudes toward implementation. Perhaps this is because it is a framework which was designed by combining aspects of many frameworks; it may be used because it is deemed to be comprehensive [25]. RE-AIM was used solely for evaluation of adoption, likely because it is accessible and easily understandable [58, 60, 73]. Few frameworks were validated or implemented. This may be due to their qualitative and theoretical basis, rather than a protocolised framework, which could be more easily quantitatively evaluated for validation. A bibliometric analysis identified that the academic impact of frameworks varied greatly, with UTAUT, CFIR, RE-AIM, NASSS and TDF garnering many more citations than others, despite other frameworks being more comprehensive (e.g. BEAR).

Due to the existence of multiple overlapping frameworks, academics and CDSS developers may choose to develop a new framework, rather than implement existing frameworks. This phenomenon could inevitably lead to further lack of standardisation, and repetition (and possible waste) of academic effort. In order to prevent this, we have included a catalogue of existing frameworks for CDSS implementation (Fig. 4). The catalogue reiterates two key findings of this study: that the most highlighted domains relate to ‘Acceptance and Integration’ phases of CDSS implementation, while the least discussed domains relate to CDSS ‘Design and Development’. The catalogue will be useful to CDSS co-developers and project teams to identify relevant guidance to consider at each stage of implementation. Other authors have asserted that multiple frameworks may be more appropriate than a single comprehensive one, to address each aspect of the development-to-adoption process of CDSS implementation [88]. We agree, and offer this guide to frameworks, rather than attempt to combine all domains into a single framework. Many CDSS studies use existing frameworks rather than creating new frameworks, such as CFIR and PRISM. Or, the frameworks are often used together, such as an implementation framework (e.g. CFIR, PRISM or RE-AIM) to capture the breadth of contextual issues, alongside a technology framework (e.g. UTAUT), which provides more granular insight on socio-technical issues. This work augments existing online resources for understanding and choosing implementation frameworks [89].

There are some limitations to this study. It is possible that our search strategy missed out on relevant papers. Even if this is a representative sample of papers published in both medical and artificial intelligence (AI) journals and conference proceedings, it may not reflect the entire range of the literature on CDSS adoption frameworks. It is also possible that a small number of relevant papers were not included because they did not use the selected keywords in their title or abstract, though we mitigated this by also searching references of references and expert recommendations. Another limitation is the subjectivity with which the domains and sub-domains were grouped and mapped across the implementation stages from the four reviewers. We mitigated this by ensuring two reviewers reviewed and extracted data from each paper: one non-medical researcher / computer scientist and one clinician, and consensus was achieved in case of a disagreement. This process took time, and therefore a further limitation is that publications published in 2022 or early 2023 were not included. Further because we specified a time period of 2000–2021 in our search strategy, some well-known frameworks for utility and adoption of technology (such as UTAUT) were not included in the first research objective, though they were captured by the second objective. Another limitation to the framework use section is that frameworks could only be identified as being in use if it was published in an academic journal and met inclusion criteria for our study. This was partially mitigated by the bibliometric sensitivity analysis. However, there may be frameworks in use without being published in an academic journal, and academic citations may not always denote positive impact; frameworks may be cited by authors to describe negative aspects (e.g. to highlight bias or their inadequacies as a framework). Future research directions may include more work into less-studied aspects, especially design and development of CDSS frameworks. Further, few frameworks have been formally validated, which would help teams choose whether and how these complex interventions should be applied. An emphasis on collaboration between clinician and non-clinician affected parties may help advance the field.

## Conclusions

Many CDSS implementation frameworks exist. However, for co-developers, choosing relevant frameworks may be a challenge. A taxonomy of domains addressed by CDSS implementation frameworks is provided, as well as a description of their use, and a catalogue of frameworks listed by the implementation stages they address. The most frequently published implementation stage is ‘acceptance and integration’ of CDSS, while ‘design and development’ is the least-studied. More effort should be placed on framework validation, and engaging with key affected parties of CDSS implementation, including clinicians, developers, non-medical researchers / computer scientists, and patients.

## Data Availability

Not applicable.

## Abbreviations

CDSS: Clinical decision support systems
IC: Inclusion criteria;
EC: Exclusion criteria EC
JW: Jared Wohlgemut
RS: Rebecca Stoner
EP: Erhan Pisiris
EK: Evangelia Kyrimi
RQ: Research questions
PRISMA-ScR: Preferred reporting items for systematic reviews and meta-analyses extension for scoping reviews
M: Medical clinicians
NM: Non-medical researchers / computer scientists
B: Both medical clinicians and non-medical researchers / computer scientists
O: Other
N: New framework
E: Extension to an existing framework
I: Integration of an existing framework
V: Validation of an existing framework
N/A: Not applicable
BEAR: Behaviour and Acceptance Framework
iCPR: Integrated clinical prediction rule
CFIR: Consolidated Framework for Implementation Research
HITET: Health Information Technology Evaluation Toolkit
UTAUT: Unified Theory of Acceptance and Use of Technology
TTF: Task-technology fit model
HOT-Fit: Human, Organisation, and Technology-fit Framework
RE-AIM: Reach, Efficacy, Adoption, Implementation, and Maintenance framework
NPM: Normalization process model
NASSS: Nonadoption, abandonment, scale-up, spread, and sustainability
GRASP: Grade and Assess Predictive tools
TPOM: Technology, People, Organizations, and Macroenvironmental factors
MRC: Medical Research Council
HITREF: Health Information Technology (HIT) Reference-based Evaluation Framework
IDEAS: Integrate, Design, Assess, and Share
PRISM: Practical Robust Implementation and Sustainability Model
TDF: Theoretical Domains Framework
UASAD: The user acceptance and system adaptation design model
IPOE: The input-process-output-engage model
GUIDES: Guideline Implementation with Decision Support
ISO: International Organisation for Standardisation
